# Hearing rehabilitation of children and adolescents with unilateral hearing loss

**DOI:** 10.1590/2317-1782/20212021065

**Published:** 2022-12-05

**Authors:** Ângela Leusin Mattiazzi, Ana Clara de Lima Malheiros, Julia Dalcin Pinto, Iara Denise Endruweit Battisti, Eliara Pinto Vieira Biaggio

**Affiliations:** 1 Programa de Pós-graduação em Distúrbios da Comunicação Humana, Universidade Federal de Santa Maria – UFSM - Santa Maria (RS), Brasil.; 2 Curso de Fonoaudiologia, Universidade Federal de Santa Maria – UFSM - Santa Maria (RS), Brasil.; 3 Programa de Pós-graduação em Desenvolvimento e Políticas Públicas, Universidade Federal da Fronteira Sul – UFFS - Cerro Largo, (RS), Brasil.

**Keywords:** Unilateral Hearing Loss, Child, Hearing Aids, Rehabilitation, Hearing, Perda Auditiva Unilateral, Criança, Auxiliares de Audição, Reabilitação, Audição

## Abstract

**Purpose:**

To describe the audiological characteristics and the type of intervention chosen on unilateral hearing loss cases in children and adolescents as well as to analyze correlations between the degree of hearing loss, the indication and the use of electronic devices.

**Methods:**

Observational, descriptive and retrospective study, carried out with information of 34 medical records from children and adolescents with unilateral hearing loss, assessed by two auditory rehabilitation services of medium complexity, throughout 2016 to 2019. Descriptive and Inferential statistical analysis were performed with the data.

**Results:**

A predominance of profound sensorineural unilateral hearing loss in the right ear, of pre-lingual character, with 20.6% of malformations. The most adopted intervention was the hearing aid indication, although its use is low, regardless of the degree of the hearing loss. An association was found between the degree of the hearing loss and the healthcare professionals in indicating the use of the devices.

**Conclusion:**

The indication of hearing aids is the most frequent and this decision is influenced by the degree of the hearing loss, in which the devices are mostly indicated for mild to severe losses, with bigger divergence of conduct for profound hearing losses.

## RESUMO

**Objetivo:**

Descrever as características audiológicas e o tipo de intervenção tomada em casos de perda auditiva unilateral em crianças e adolescentes e analisar correlações entre o grau da deficiência auditiva, a indicação e o uso de auxiliares de audição.

**Método:**

Estudo observacional, descritivo e retrospectivo, realizado com informações dos prontuários de 34 crianças e adolescentes com perda auditiva unilateral, atendidos em dois serviços de reabilitação auditiva de média complexidade, no período de 2016 a 2019. Realizou-se a análise estatística descritiva e inferencial dos dados.

**Resultados:**

Predomínio de perda auditiva unilateral do tipo neurossensorial, de grau profundo à direita, pré-lingual com 20,6% de malformações. A intervenção mais adotada foi a indicação de prótese auditiva, porém o uso é baixo, independente do grau da perda. Houve associação entre o grau da perda auditiva e a decisão dos profissionais em indicar o uso dos dispositivos.

**Conclusão:**

A indicação de próteses auditivas é a mais frequente e esta decisão é influenciada pelo grau da perda auditiva, sendo os aparelhos auditivos indicados para as perdas leves a severas, com uma maior divergência de condutas para as perdas profundas.

## INTRODUCTION

In the past, children with Unilateral Hearing Loss (UHL) were thought to normally develop in a way that such deficit was not seen as clinically relevant^(1)^. Most cases were considered not suitable for Hearing Aid (HA) adaptation, being the management of classroom position the only care implemented^(2)^.

New research has evidenced that among the consequences of UHL in the infant population, risk to language development has been highlighted as speech problems^(3)^, school difficulties^(4,5)^, limitations in communicative activities, especially in noisy environments^(6)^, and difficulty in sound localization^(1,7)^ have been shown in children with UHL, all of which may potentially harm language development. The implications of UHL can even go beyond those related to hearing- and language- disorders, interfering with the quality of life^(8)^ or even the intelligence scores^(9)^ of these children.

Nevertheless, one cannot state that the use of HA in cases of UHL is unnecessary^(7)^. In addition, in UHL due to malformations, the treatment possibilities include besides conventional HA by airway or bone conduction, bone anchored hearing aids or surgical reconstruction of the ear^(10)^.

Other electronic devices, such as the Contralateral Routing of Signal (CROS), the Modulated Frequency system (FM system) and the Cochlear Implant (CI)^(2,11)^, have also been reported by literature as options to adapt UHL cases. The latter, however, does not present proven scientific evidence of its benefits in childhood^(2)^.

Recently, remarkable attention has been drawn to UHL rehabilitation due to factors such as the ever since decreased age of diagnosis as well as the results of studies highlighting the impacts of UHL on children's language and learning^(1,3-5)^ . Thus, the analysis of how the auditory rehabilitation services are managing the cases of children with UHL is a relevant topic considering the plurality of conducts observed in the scientific literature.

In Brazil, the rehabilitation of UHL is envisaged by the National Policy to Hearing Health Attention, a measure adopted by the Sistema Único de Saúde (SUS)^(12)^ since 2004, of which includes the granting of HA and speech therapy. As for UHL cases in particular, the suitability to adapt HA, according to the instructions provided^(13)^, is controversial and requires a justification so that the HA can indeed be concessed, e.g. difficulty in social integration. The aim of the therapeutic intervention in these cases is to stimulate auditory and linguistic abilities centered on family participation, relying on specific therapeutic strategies depending on the individual demands of each case^(14,15)^.

In view of the above, the aim of this study was to describe the audiological characteristics and the type of intervention chosen in the cases of children and adolescent with UHL attended by auditory rehabilitation public services, as well as to analyze the correlations between the degree of hearing loss, the indication and the use of HA.

## METHODS

This is an observational, descriptive and retrospective study, carried out with information from the medical records of two medium complexity hearing rehabilitation services of the SUS, both located in the state of Rio Grande do Sul.

137 medical records of children and adolescents were analyzed throughout january 2016 to november 2019. Of these, 34 (24,8%) were found to have UHL, composing the sample of the study. The age range was 11 months to 18 years, mean age of 8.7 years, being 18 males and 16 females. The records were excluded only if there was any lacking information or incomplete data.

The analyzed variables were: type and degree of hearing loss, affected ear, age of diagnosis, age of onset for hearing loss, use of HA and effectiveness of its use, image exams and speech therapy.

The UHL cases were classified according to the type of hearing loss in one of the three categories: sensorineural, conductive or mixed^(16)^. To define the degree of hearing loss, the already established classifications of both services were used, which differed according to the age of the subject. For children older than seven years old, the classification proposed by Lloyd and Kaplan^(17)^ was used and for those younger, Northern and Downs^(18)^ was used. Both information were extracted from the most recent audiological assessment available in the subject’s record. Both classifications on the hearing losses’ degree were used as this study portrays an analysis of medical records, thus, the authors decided to maintain the classification in order to respect the decision-making of the rehabilitation services studied.

The information regarding the use of HA, the indication and the type were verified in the otorhinolaryngological record. To address the use effectiveness of the devices, a total of eight or more hours was considered as effective. The information on this matter was examined through analyzing the data logging of the software of the device or in the last update in the medical record.

The age of onset of UHL (pre- or post-lingual) and the presence of image testing were considered according to the otorhinolaryngological record. Only two subjects had imaging tests and the others had clinically proven malformations (microtia and atresia or agenesis of the external auditory canal) after medical consultation. It’s important to highlight that further malformations in the remaining subjects of the study could only be discarded with imaging testing (Magnetic Resonance Imaging and/or Computerized Tomography of Maistoid). However, such exams were not requested and/or were not available at the services that received these subjects.

Regarding the speech therapy, information related to whether the participants were exposed or not to therapeutic services as well as the main goals of the assistance were sought in the same records. The relation between the speech therapy method and the auditory abilities was not analyzed in this study.

After the data were collected, a series of descriptive and inferential statistical analyses were performed. The correlation between the degree of hearing loss and the hours of HA use was measured using Spearman's correlation. Lastly, Fisher's exact test was used to verify the association among the decision of indicating the use of electronic devices, the degree of hearing loss and the age of diagnosis. A 5% level of significance was considered to all statistical tests, which were performed in the software R v.2.15.3.

This study was approved by the Ethics in Research Committee of the institution of origin under the number 14804714.2.0000.5346 and all the ethical precepts established by Resolution No. 466/2012 of the National Health Council (NHC) that regulates research involving human beings were respected. Those responsible for the patients, when accessing the rehabilitation services, signed the Term of Acceptance to the Service Norms as well as a written informed consent terms, which states that all the medical record data may be accessed by the professionals of the service in a confidential manner, and can therefore be used for research.

## RESULTS

Sensorineural UHL was the most frequent type observed in 22 (65%) of the subjects, followed by conductive hearing loss, evidenced in eight (23%) subjects and mixed UHL was observed in four (12%) subjects. From the 34 children and adolescents included, 23 (67,6%) had the right ear affected while 11 (32,4%) exhibited UHL in the left ear. In regard to the age of onset of UHL, a pre-lingual character was identified in 23 (67,6%) subjects and post-lingual in 11 (32,4%).

**Figure 1:**
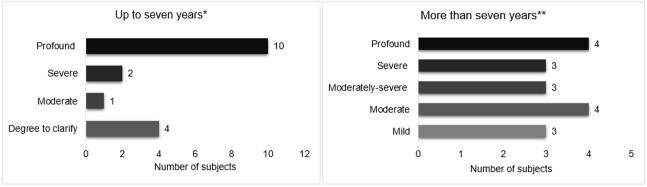
Degree of unilateral hearing loss in children and adolescents (n = 34); * = Classification of the degree of hearing loss according to Northern and Downs (2002); ** = Classification of the degree of hearing loss according to Lloyd and Kaplan (1978)

Figure 1 shows the distribution of the sample in regards to the degree of hearing loss.

From the studied sample, seven (20.6%) subjects were found to have malformations involving the ear. Table 1 depicts the age, description of the type of anomaly and the presence of image testing as well as the conduct adopted in these cases.

**Table 1:** Association between the decision to indicate the use of electronic devices and the degree of hearing loss (n=18)

	**Electronic device**		
**Degree of hearing loss**	**With indication (%)**	**Without indication (%)**	**p** #
Mild	66.7	33.3	0.037*
Moderate	100.0	0.0	
Moderately severe	66.7	33.3	
Severe	75.0	25.0	
Profound	42.9	57.1	

* Statistically significant value

# Fisher's Exact Test

The age of the subjects at the moment of the audiological diagnosis varied between one month to 17 years, mean of 5.2 ± 5.1 years (mean ± standard deviation). 12 (35.3%) cases were identified in the Newborn Hearing Screening (NHS) and for these, the diagnosis occurred in the average of 3.3 months.

**Figure 2:**
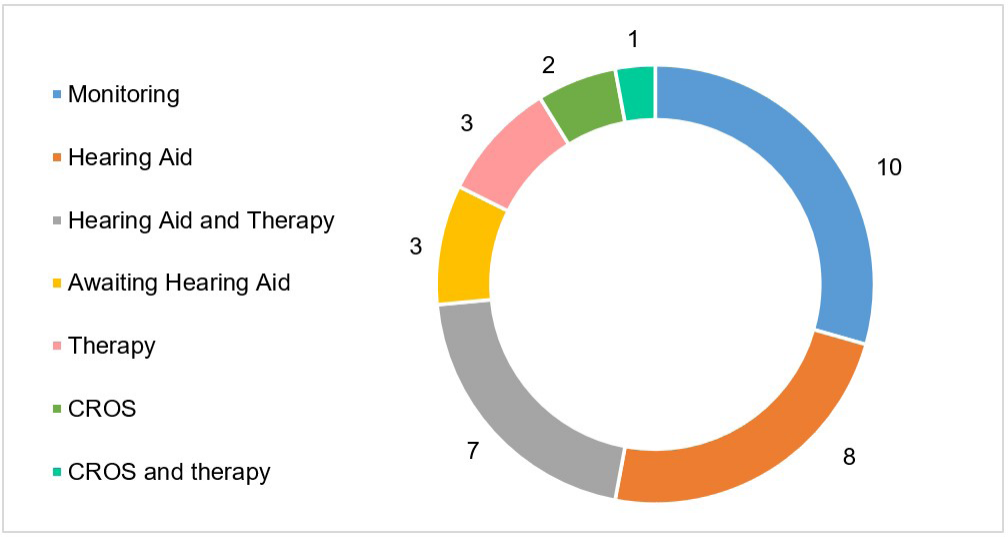
Type of intervention adopted for unilateral hearing loss in children and adolescents (n = 34); Hearing aids: individual sound amplification device; Caption: CROS = Contralateral routing of signal

The distribution of the subjects in regards to the adopted intervention can be observed in Figure 2.

It’s noteworthy that all subjects were under audiological monitoring to monitor a possible progression of the hearing loss and/or involvement of the contralateral ear, but in 10 cases this was the only decision made as a conduct.The use of HA was indicated for most of the participants. HAs were indicated for 15 subjects (83,3%) while three (16.7%) received the indication of CROS.

In regards to speech therapy, 11 (32.3%) children and adolescents performed this intervention weekly. Seven cases were found to use HA and also attend weekly speech therapy, of which had the specific aims to train auditory abilities, stimulate speech/language and sensibilize family to ensure adherence and raise awareness of the HA use as well as the therapeutic process. The therapeutic aims for the three children that did not use HA but were undergoing speech therapy was to stimulate the speech/language development as well as the development of the auditory abilities of the unaffected ear. No information on the therapeutic aims was found in the medical records of the subjects who used CROS and were also attending speech therapy. The reasons why the rest of the subjects did not attend speech therapy sessions varied, according to their medical records, from presenting satisfactory evolution in terms of auditory and/or linguistic abilities or they lived far away from the auditory rehabilitation services in which this study was carried out.

The information regarding the average hours of use of the electronic devices (datalogging) was found in only 14 medical records. The amount of hours of use varied between 0.3 hours to 11.8 hours per day, a mean of 4.5 ± 2.9 hours (mean ± standard deviation), for both HA and CROSS.

The Spearman correlation did not evidence significant correlation between the degree of hearing loss and the hours of use (datalogging) (p=0.327).

Also, Table 1 shows the association between the decision of the healthcare professionals in indicating the use of electronic devices and the degree of hearing loss. Both variables were found to be associated (p=0.037).

The Fisher’s exact test was employed to analyze the association between the decision of the healthcare professionals in indicating the use of electronic devices and the age of diagnosis, although no statistically significant difference was obtained for this analysis (p=0.140). The cut-off point of seven years was considered for this analysis.

## DISCUSSION

By analyzing the data of the present study, we found that children and adolescents present predominantly profound sensorineural UHL in the right ear. The most opted conducted was the indication of HA, although the use of such devices were found to be low. Moreover, the decision of the healthcare professionals to indicate such devices is apparently influenced by the degree of hearing loss.

Sensorineural hearing loss was the most frequently observed type of UHL, followed by conductive and, lastly, mixed. Profound hearing loss was evidenced in 41.2% of the sample. Other studies^(1,4,9,19)^ have also observed higher prevalence of profound sensorineural hearing loss in the infant population with UHL. A study showed worse results in speech and language assessments of children with severe to profound UHL when compared to other degrees of losses^(20)^. Literature points that when the auditory deficit is higher, the deprivation of sensory stimuli in the auditory pathways tends to be higher as well and consequently, areas such as speech and language end up more compromised due to these aspects. Therefore, the degree of the hearing loss appears to clearly influence the language development of the subjects with UHL^(20)^.

In regard to the affected ear, a predominance of the right ear was observed in the present study, just like in the consulted literature^(1,3,10,21)^. The same predominance was also evidenced for malformation cases^(10)^. It's important to consider that, in UHL cases, a shorter activation of the contralateral auditory cortex occurs, therefore, when it comes to right UHL, the left auditory cortex tends to be more deprived and so a higher risk to speech recognition and auditory localization may be expected ^(2)^. A Brazilian research carried out in the state of São Paulo found that children with right UHL showed more school and linguistic complaints than those with left UHL^(22)^.

A higher occurrence of referral to high complexity otologic services was observed in the children with UHL due to malformations in the present study (Chart 1). Such referral had the objective of assessing the surgical possibilities to reconstruct the ear or to adapt bone anchored HA, as such options were not available in the services where this study was performed. Literature indicates that the conduct of surgical reconstruction is frequent, although it does not always guarantee good prognosis results^(23)^. In some cases, the use of HA is necessary as normal hearing is hardly obtained even after the surgical reconstruction^(10)^.

**Chart 1:** Age, anomaly, imaging and conduct of children and adolescents with unilateral ear malformations (n=7)

**Subject**	**Age**	**Anomaly**	**Image Testing**	**Conduct**
1	3	Microtia and WEM agenesis	No	Referred to high complexity.
2	2	Microtia and WEM atresia	No	Hearing aids and speech therapy
3	3	Microtia and WEM Atresia	Yes	Referred to high complexity and speech therapy
4	4	Microtia and WEM agenesis	Yes	Referred to high complexity and speech therapy.
5	2	Microtia and WEM agenesis	No	Referred to high complexity and audiological follow-up.
6	3	Microtia and WEM Atresia	No	Referred to high complexity.
7	13	WEM atresia	No	Hearing aids and speech therapy

Caption: WEM = Wide external meatus

Still on the topic of ear-related malformations, bone anchored hearing devices such as the Bone Anchored Hearing Aid (BAHA) may also be a good alternative^(23)^. It’s necessary to discard all the adaptation possibilities of airway HA and thus, the investigation of the anatomical conditions of the external ear becomes more relevant. In this sense, audiological assessment may not be sufficient in these cases, requiring further exams such as image testing^(10)^. In Chart 1, we can observe that only two subjects had already been submitted to image testing. This fact can be justified by the limited access available in the Brazilian public healthcare system of complementary assessment of such kind.

It's important to emphasize that external ear malformations are generally associated with middle ear malformations and that, in 15% to 20% of the cases, internal ear malformations coexist with those of external ear as well. The justification for such fact may be found in the embryonary development, as the external and middle ear present the same embryonic origin while the internal ear develops separately in a previous gestational period^(24)^.

In this study, the identification of the hearing loss occurred mostly in the preschool period, considering the mean age of diagnosis was 5.2 years, which is in agreement with other Brazilian study^(22)^. Literature believes that UHL is diagnosed later than bilateral hearing losses as they may appear imperceptible to others, such as the parents, for a higher period of time^(25)^. Attention must be drawn to the 12 cases identified by the NHS, of which the diagnosis occurred on average at 3.3 months. This result in particular shows an important decrease of the age of diagnosis recommended by the international guidelines.

A high clinical variability was found in regards to the adopted intervention when it comes to auditory rehabilitating patients with UHL and, therefore, one can infer that such cases represent a challenge to doctors and audiologists, which is more evidenced when the target-public is of children^(26)^. As observed in Figure 2, the majority of the sample (52.9%) received indication of electronic devices, which for 15 (44.1%) the HA was indicated and the CROS was chosen for three (8.8%) of them. No records of FM system or CI use was found for any of the subjects. This data is in line with another study which verified that from a total of 50 children, 20 (40%) used HA and less than half of these had the FM system. The authors affirm that children with UHL may perceive the benefits of the use of the HA which negatively affects the use of complementary devices and that the rehabilitation services must consider them as options at the moment the conduct is chosen and in the family counseling as well. This is also emphasized by other study^(7)^.

Although the CROS can be indicated for children with severe to profound UHL, according to the international guidelines, evidence of its benefit is still lacking in literature for this population. Such concern is due to the fact that the presence of noise in the contralateral ear in environments such as the school could interfere in the normal ear and thus negatively affect hearing for these children^(2)^. Another negative aspect related to the use of CROS is the decreased sound localization ability, although a possible benefit of this device would be to improve speech perception in silence or in situations where noise and speech are presented at front^(27)^.

As for the intervention possibilities in the UHL cases in children, the option of submitting the subjects only to the audiological monitoring in the present study was found to be expressive (Figure 2). The aim of this follow-up is to verify the progression of the hearing loss, the possible evolution to a bilateral loss, the presence of conductive impairment in the normal ear and the language/speech development^(1,4)^. The fact that the UHL can become bilateral emphasizes the importance to closely monitor this population, as about 40% of these children may present hearing loss progression, both for the affected ear and the normal-hearing ear as well^(1)^.

Another result evidenced in this research as well as in further studies on the topic^(5,28)^ refers to the time of daily usage of the electronic devices, both for HA and CROSS, which was very low. The main barriers regarding the use of HA in younger populations with UHL are: disconfort, poor perceiving of its benefits and social stigma. The use of FM system and preferential seats in classrooms appears to intensify furthermore the social stigma around children and adolescents with UHL^(9)^.

No correlation between the degree of the hearing loss and the amount of hours of use of the electronic devices was found in the studied sample. Thus, it's not possible to affirm that the degree of hearing loss influences whether the children would use more or less the HA or the CROS, as it has been previously referenced in literature^(9)^. The use of two classifications on the hearing loss degree, used in the studied services and, also, the heterogeneity of the sample could perhaps have compromised the analysis for this variable, thus we can consider these the limitations of this study.

As observed in Table 1, an association between the healthcare professionals' decision in indicating the use of electronic devices and the degree of hearing loss was evidenced. In this way, one can infer that in the cases of children and adolescents with mild to severe UHL, the professionals feel more secure to indicate HA. The same is not observed when the degree of the hearing loss is profound. This difference regarding the conduct may occur as there is a series of intervention possibilities available to the profound UHL cases, such as the CROS, the bone anchored devices and even the CI, although all of them still require further investigation to prove their benefit to the infant population^(2,9,11)^. In fact, the Joint Committee on Infant Hearing^(15)^, in its last update emphasizes the necessity to include UHL in future expansions of the criteria for CI indication. Also, due to the inexistence of protocols to verify and validate the HA, especifically for UHL in children, the healthcare professionals are unable to assess the real benefit of this particular device in the various degrees of UHL, and thus its indication gets compromised.

In the future, it would also be necessary that the Brazilian Public Services attempt to unify the assessment methods so that the conducts can be defined more properly and at an earlier stage^(6)^. Also, partnerships with HA companies would be a good possibility for these services in a way that the devices such as the HA, the CROS and FM system could be loaned for household testing and thus assist the healthcare professionals in decision-making^(19)^.

## CONCLUSION

The type and degree of UHL in children and adolescents is predominantly profound, sensorineural, pre-lingual and affects the right ear, with presence of malformations in 20.6% of the cases. The most adopted intervention was the use of HA, although the use of such devices were found to be low, regardless of the degree of hearing loss. The decision to indicate electronic devices is influenced by the degree of hearing loss, being most frequently indicated in cases of mild to severe UHL. The conduct of professionals in cases of profound UHL is divergent, which reflects the lack of guiding protocols to help decision making in the management of these cases.
